# Adsorptive interaction of bisphenol A with mesoporous titanosilicate/reduced graphene oxide nanocomposite materials: FT-IR and Raman analyses

**DOI:** 10.1186/1556-276X-9-462

**Published:** 2014-09-03

**Authors:** Chinh Nguyen-Huy, Nayoung Kim, Thuy-Duong Nguyen-Phan, Ik-Keun Yoo, Eun Woo Shin

**Affiliations:** 1 School of Chemical Engineering, University of Ulsan, Daehakro 93, Nam-gu, Ulsan 680-749, South Korea; 2 Chemistry Department, Brookhaven National Laboratory, Upton, New York 11973, USA

**Keywords:** Bisphenol A, Graphene oxide, Mesoporous titanosilicate, Adsorption sites, Interaction

## Abstract

Nanocomposite materials containing graphene oxide have attracted tremendous interest as catalysts and adsorbents for water purification. In this study, mesoporous titanosilicate/reduced graphene oxide composite materials with different Ti contents were employed as adsorbents for removing bisphenol A (BPA) from water systems. The adsorptive interaction between BPA and adsorption sites on the composite materials was investigated by Fourier transform infrared (FT-IR) and Raman spectroscopy. Adsorption capacities of BPA at equilibrium, *q*_*e*_ (mg/g), decreased with increasing Ti contents, proportional to the surface area of the composite materials. FT-IR observations for fresh and spent adsorbents indicated that BPA adsorbed onto the composite materials by the electrostatic interaction between OH functional groups contained in BPA and on the adsorbents. The electrostatic adsorption sites on the adsorbents were categorized into three hydroxyl groups: Si-OH, Ti-OH, and graphene-OH. In Raman spectra, the intensity ratios of D to G band were decreased after the adsorption of BPA, implying adsorptive interaction of benzene rings of BPA with the sp^2^ hybrid structure of the reduced graphene oxide.

## Background

Endocrine-disrupting chemicals (EDCs) are substances that mimic natural hormones in the endocrine system causing adverse effects on humans and wildlife [1,2]. EDCs are considered to be exogenous agents that interfere with the synthesis, secretion, transport, binding, action, and elimination of natural hormones in the body responsible for the maintenance of homeostasis, reproduction, development, and behavior. Exposure to EDCs could have a substantial effect on the body, disrupting bodily functions and processes. Bisphenol A (BPA), an EDC, is a cause of considerable social and scientific concern. It is heavily used as a monomer in the synthesis of epoxy resins and polycarbonate plastics [3] and is considered to be a significant pollutant since its weak estrogen-like effect is harmful to organisms [3-5]. Various diseases (including carcinogenesis) may result from exposure to BPA. In recent years, BPA has been detected in industrial wastewater, groundwater, surface water, and drinking water [6-9].

Various technologies have been attempted to remove BPA from water systems, such as adsorption [10-12], biological treatment [13], and photodegradation technology [14]. Among these methods, adsorption is a superior and promising method for removing low-concentration contaminants from water systems in terms of cost, ease of operation, and lack of harmful secondary products. The removal of pollutants by the adsorption process depends on the inherent physicochemical properties of the pollutants and adsorbents.

Graphene, a single sp^2^-hybridized carbon layer arranged in a honeycomb structure, is currently one of the most exciting new materials due to its large surface area, excellent electronic and mechanical properties, and good thermal conductivity [15-17]. The remarkable properties of graphene allow it to be applied in many research fields, including adsorbents. Mesoporous silicate has been used for environmental applications because of its large surface area and interconnected channels [18,19]. Ti has been incorporated into the framework of mesoporous silicate to significantly improve its hydrothermal stability, adsorbability, and photocatalytic performance [18-21]. Recently, the combination of graphene or reduced graphene oxide nanosheets and TiO_2_ particles has been shown to be a promising candidate for both adsorption and photocatalysis in water systems owing to the improved accessibility to pollutants and efficient facilitation of charge carrier separation [22-25].

In this work, mesoporous titanosilicate/reduced graphene oxide composite materials were synthesized as a function of Ti content, and their adsorptive characteristic for BPA in an aqueous solution was investigated through FT-IR and Raman spectroscopy for the first time. FT-IR analysis showed that the hydroxyl groups on the composite materials interacted with BPA to produce hydrogen bonding and the hydroxyl groups in the mesoporous titanosilicate could be divided into two different types - Si-OH and Ti-OH. In addition, Raman spectra gave evidence that the sp^2^ hybrid structure of graphene oxide interacted with the benzene rings of BPA. This work may provide new insights into adsorptive interactions between the adsorption sites of inorganic/organic composite materials and organic contaminants.

## Methods

### Preparation of composite materials

All chemicals were obtained from Sigma-Aldrich Korea (Yongin, Kyunggi, South Korea) and were used as received without any purification. In a typical procedure, graphene oxide (GO) was synthesized from expanded graphite (grade 1721, Asbury Carbons, Asbury, NJ, USA) by a modified Hummers method [26]. For the preparation of mesoporous titanosilicate/reduced graphene oxide composite materials, an aqueous solution containing cetyltrimethyl ammonium bromide (CTAB) and NaOH was mixed until it became homogeneous; then, the GO dispersion was added (approximately 10 wt%). The mixture was sonicated for 2 h at room temperature and then magnetically stirred at 313 K for 2 h. A mixture of tetraethyl orthosilicate and titanium isopropoxide (various Ti:Si molar ratios were used for different compositions) was added drop-wise. The whole solution was stirred at 313 K for 12 h and then hydrothermally treated at 353 K for 10 h. Solid products were recovered by washing with warm ethanol and drying at 353 K overnight. The as-synthesized powders were finally heat-treated at 823 K for 4 h under flowing Ar (15 ml/min). The calcined materials were denoted as ‘**MTSG-i**’ for mesoporous titanosilicate/graphene composite materials, where ‘i’ is the Ti content (1, 5, 10, and 20 wt%).

### Characterization techniques

Fourier transform infrared (FT-IR) spectra were obtained on a Nicolet 380 FT-IR spectrometer (Thermo Electron Co., Waltham, MA, USA) using the KBr pellet technique. Raman spectra were collected using a DRX Raman microscope (Thermo Fisher Scientific, Waltham, MA, USA) with a 633-nm laser excitation. N_2_ sorption measurements were conducted on a Micromeritics ASAP 2020 instrument (Micromeritics, Norcross, GA, USA). The samples were degassed under a vacuum at 240°C for 5 h prior to automatic analyzer analysis at −196.15°C. The Brunauer-Emmett-Teller (BET) calculation method was applied to determine the specific surface area.

### BPA adsorption

The adsorptivity of the composite materials was elucidated through the removal of BPA in water. Specifically, 50 mg of the adsorbent was immersed in 100 ml of the BPA-containing solution (initial concentration = 10 mg/l) under constant stirring inside a dark chamber. The pH of the solutions was 6.0 ± 0.3. The concentration of the BPA solution before and after adsorption at room temperature was measured by a UV-vis absorbance microplate spectrophotometer (SpectraMax® Plus 384, Molecular Devices, Sunnyvale, CA, USA). The amount adsorbed onto the adsorbent, *q*_*e,exp*_ (mg/g), was calculated by a mass balance relationship:

(1) $$q_{e,\exp}=\left(C_{0}-C_{e}\right)\frac{V}{W}$$

where *C*_*0*_ and *C*_*e*_ are the initial and equilibrium liquid-phase concentrations of BPA (mg/l), *V* is the volume of the solution (l), and *W* is the weight of the dry composite materials used (g).

After adsorption tests, spent adsorbents were then filtered using a 0.22-μm GV filter, dried inside a hood, and wrapped in aluminum foil. Dried spent adsorbents were used for Raman and FT-IR analyses for observing the adsorption interaction between BPA and the adsorbents.

## Results and discussion

### Adsorption behavior

**Figure 1:**
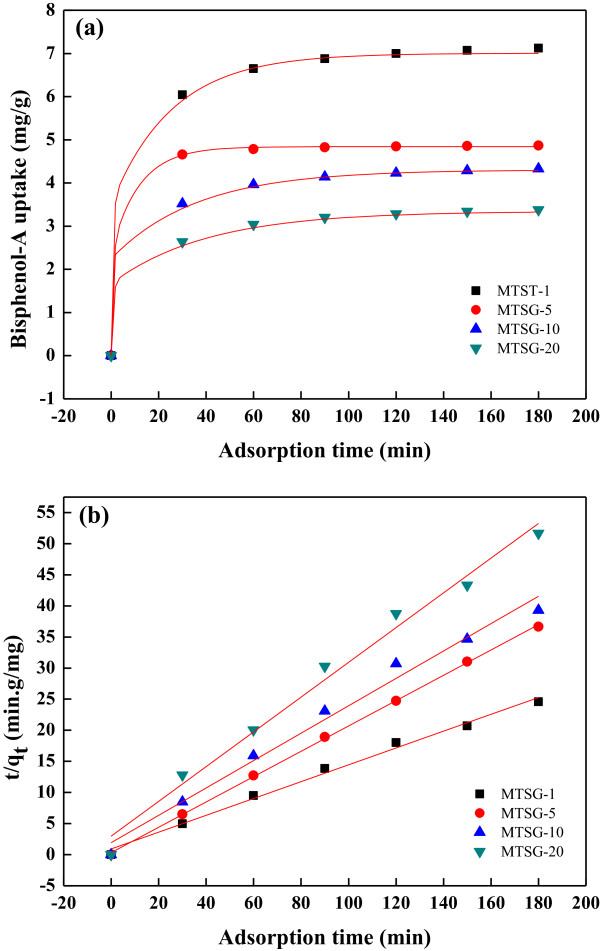
**Adsorption behavior of MTSG-i adsorbents for BPA. (a)** Adsorption kinetic data and **(b)***t*/*q*_*t*_ vs. *t* plots for the fit of the pseudo-second-order kinetic model.

The adsorption behavior of MTSG-i for BPA was monitored by adsorption kinetics shown in Figure 1. With increasing Ti content, adsorption amounts at equilibrium (*q*_*e*_) increased. To quantitatively measure the adsorption behavior, a pseudo-second-order kinetic model was employed, resulting in *q*_*e*_ values and an adsorption rate constant (*k*_*ads*_) from the fit of the adsorption kinetic data. Herein, the pseudo-second-order equation is in the form [27]:

(2) $$\frac{dq_{t}}{\mathrm{dt}}=k_{\mathrm{ads}}{\left(q_{e}-q_{t}\right)}^{2}$$

where *q*_*e*_ and *q*_*t*_ are the adsorption capacity at equilibrium and at time *t*, respectively (mg/g_catalyst_) and *k*_*ads*_ is the rate constant of pseudo-second-order sorption (g_catalyst_/mg *·* min). By integrating Equation 2 for the boundary conditions (*t* = 0 to *t* = *t* and *q*_*t*_ = 0 to *q*_*t*_ = *q*_*t*_), the following linear form can be written as:

(3) $$\frac{t}{q_{t}}=\frac{1}{kq_{e}^{2}}+\frac{1}{q_{e}}t$$

**Figure 2:**
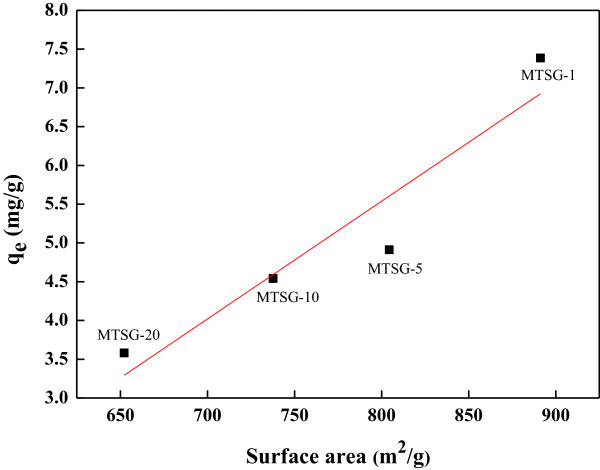
**A plot of adsorption amount at equilibrium (*q***_***e***_**) for BPA vs. surface area of MTSG-i adsorbents.**

In the present study, the experimental data evaluated from the linear transform (*t*/*q*_*t*_) = *f(t)* conform to the pseudo-second-order adsorption kinetics. Therefore, the *q*_*e*_ and *k*_*ads*_ values are determined from the slope and intercept of the plot. The *q*_*e*_ and *k*_*ads*_ values obtained from the fits are listed in Table 1. Fitted *q*_*e*_ values are a little bit higher than the *q*_*e,exp*_ values, but show the same trend for the Ti content, namely, that the values decrease with increasing Ti content. In the literature, Ti incorporation into the framework of mesoporous silicates enhanced hydrothermal stability, adsorbability, and photocatalytic performance [18,23]. However, it also induced a decrease in surface area with increased Ti loading [19]. In this study, with the Ti incorporation, the surface areas of the composite materials decreased (Table 1). In Figure 2, the *q*_*e*_ values are plotted along with the surface area of the composite materials. It clearly shows that the *q*_*e*_ value is proportional to the surface area of the composite materials. Accordingly, the surface area of the composite materials is a key parameter for BPA adsorption, and the decrease in the *q*_*e*_ value of the composite materials is caused by a reduction in the surface area by Ti addition.

**Table 1:** **Adsorption amount at equilibrium (*q***_***e***_**) and coefficient of the pseudo-second-order model for BPA adsorption**

**Sample**	**Experimental *q***_**e,exp **_**(mg/g**_**ads**_**)**^***a***^	**Adsorption efficiency (%)**^***b***^	***k *** **(g**_**ads**_**/mg** ***·*** **min)**^***c***^	***q***_***e ***_**(mg/g**_**ads**_**)**^***c***^	***R***^***2 ***^**value**^***c***^	**Surface area (m**^**2**^**/g)**^***d***^
MTSG-1	6.6488	36.6	0.0203	7.386	0.9934	891.3
MTSG-5	4.8821	24.6	0.1263	4.912	0.9996	804.5
MTSG-10	3.9987	22.9	0.0254	4.539	0.9863	737.8
MTSG-20	3.4748	17.4	0.0262	3.579	0.987	652.2

^*a*^Equilibrium adsorption capacity obtained from the kinetics experiment; ^*b*^adsorption efficiency at the equilibrium state; ^*c*^adsorption rate constant, equilibrium uptake, and correlation coefficient determined from the pseudo-second-order adsorption model (*t*/*q*_t_) *vs. f*(*t*); ^*d*^measured from N_2_ adsorption/desorption.

### Adsorptive interaction of BPA with the composite materials

**Figure 3:**
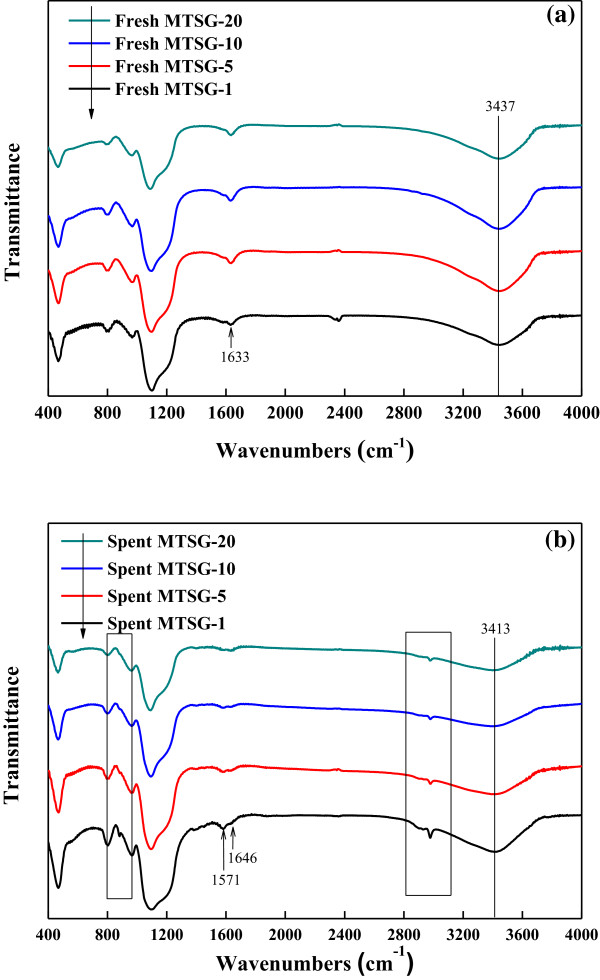
FT-IR spectra of (a) fresh MTSG-i and (b) spent MTSG-i adsorbents.

FT-IR spectra for fresh and spent adsorbents were collected to analyze the adsorptive interactions of BPA. Figure 3a,b shows FT-IR spectra of fresh and spent MTSG-i, respectively. Primary infrared bands related to the mesoporous titanosilicate framework are the asymmetric and symmetric stretch of Si-O-Si around 1,094 and 804 cm^−1^, respectively. The band around 470 cm^−1^ is assigned to the Si-O bending vibration. Conventionally, the band around 962 cm^−1^ corresponds to the Si (or Ti)-OH vibration [28-31]. The broad bands around 3,400 to 3,450 cm^−1^ and 2,900 to 2,990 cm^−1^ represent OH and CH stretching modes, respectively. Assignment of the band at 1,634 cm^−1^ is important in this study. It can be assigned to the OH bending vibration of adsorbed water, the C = C skeleton vibration, or the C = O asymmetric stretching vibration. In this study, it should be assigned to the OH bending vibration since the intensity of the band closely correlates with that of the OH stretching vibration [28-31].

**Figure 4:**
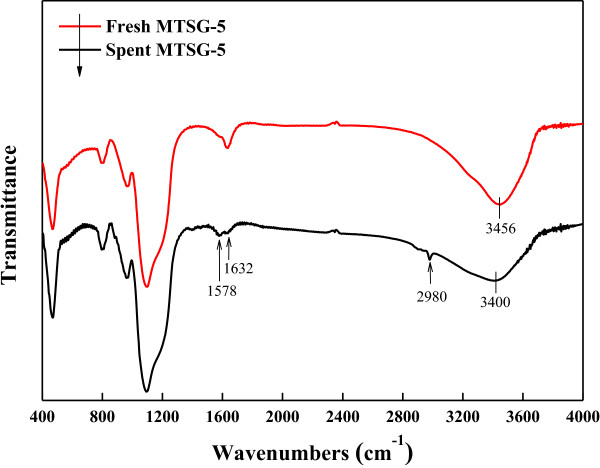
FT-IR spectra of fresh MTSG-5 and spent MTSG-5 adsorbents.

The change in FT-IR spectra after BPA adsorption is similar irrespective of the Ti content. Hence, to show the difference in FT-IR spectra by BPA adsorption in detail, FT-IR spectra of fresh and spent MTSG-5 are presented in Figure 4. The first change in FT-IR spectra as a result of BPA adsorption is the appearance of new IR bands around 2,900 to 2,990 cm^−1^ assigned to the CH stretching vibration. Those bands represent BPA adsorbed onto the composite materials. The second change in the FT-IR spectra is related to OH vibration. The band position of the OH stretching vibration is shifted from 3,448 to 3,400 cm^−1^, and the band intensities of OH stretching (3,448 cm^−1^) and bending (1,634 cm^−1^) vibrations were decreased after BPA adsorption. These phenomena indicate that OH functional groups on the composite materials interacted with hydroxyl groups contained in BPA resulting in hydrogen bonding [32].

**Figure 5:**
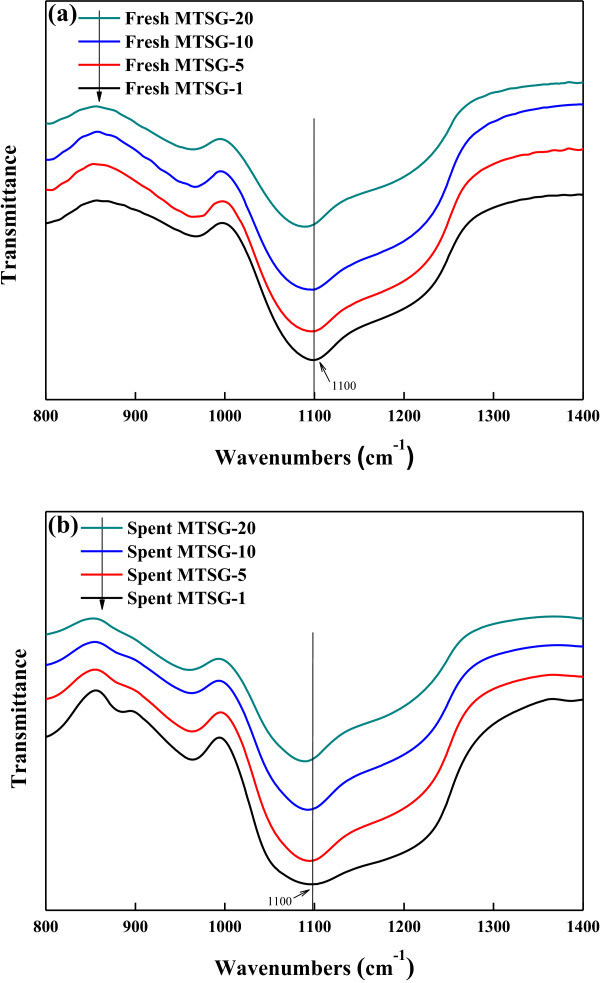
**Detailed FT-IR spectra of vibrations for the mesoporous titanosilicate framework. (a) **Fresh MTSG-i and **(b)** spent MTSG-i adsorbents.

Meanwhile, there is a change in FT-IR spectra due to the Ti content. Figure 5 shows FT-IR spectra related to mesoporous titanosilicate. In Figure 5a, the band around 1,090 cm^−1^, representing the stretching mode of Si-O-Si, moves from 1,100 to 1,088 cm^−1^ with increasing Ti content. This is also observed even for spent MTSG-i. This implies that added Ti incorporates into the framework of mesoporous silicate to make Ti-O-Si or Ti-O-Ti bonds. Replacing Si atoms in the framework with Ti atoms induces a redshift in wave number because the wave number is inversely proportional to the root of the harmonic average of atomic mass. Consequently, based on the redshift of the band around 1,090 cm^−1^, it can be concluded that there are at least two different types of hydroxyl groups even in the mesoporous titanosilicate structure: Si-OH and Ti-OH.

**Figure 6:**
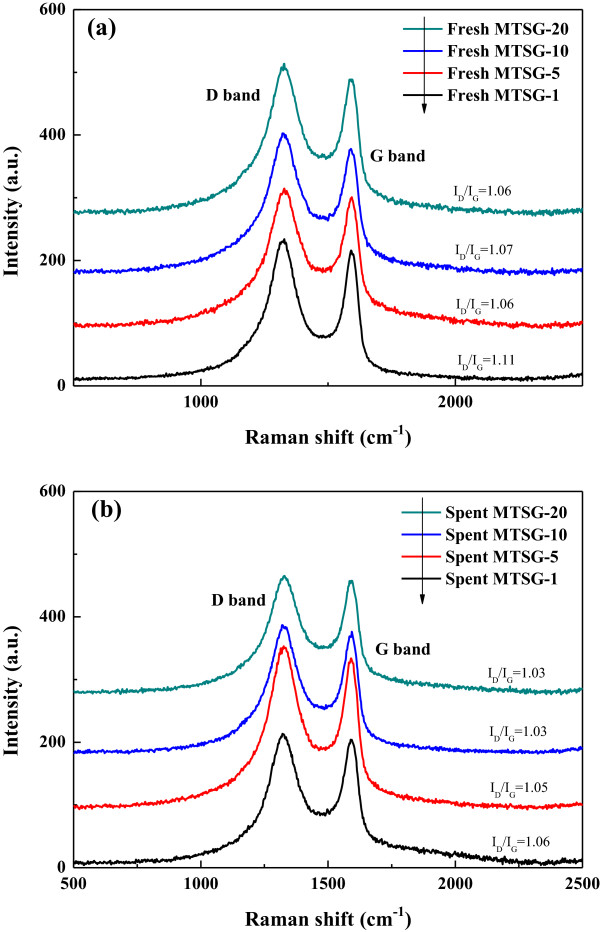
Raman spectra of (a) fresh MTSG-i and (b) spent MTSG-i adsorbents.

Raman spectra for fresh and spent MTSG-i were also collected to evaluate the adsorptive interaction between BPA and graphene oxide, shown in Figure 6. Two indicative D and G bands at 1,340 and 1,590 cm^−1^ of the composite materials are associated with the breakage of symmetry by edges or a high density of defects, and the first-order scattering of E_2g_ vibration mode observed for the sp^2^ domain, respectively [33]. The relative intensities of those broad bands for all MTSG-i decreased after BPA adsorption, illustrating either an increase in the average size of sp^2^ domains or a change in the properties of the reduced graphene oxide sheets due to interactions with BPA. Accordingly, the decrease in *I*_D_/*I*_G_ ratio by BPA adsorption implies the existence of π-π interactions between the benzene rings of BPA and the sp^2^ domains of the reduced graphene oxide.

**Figure 7:**
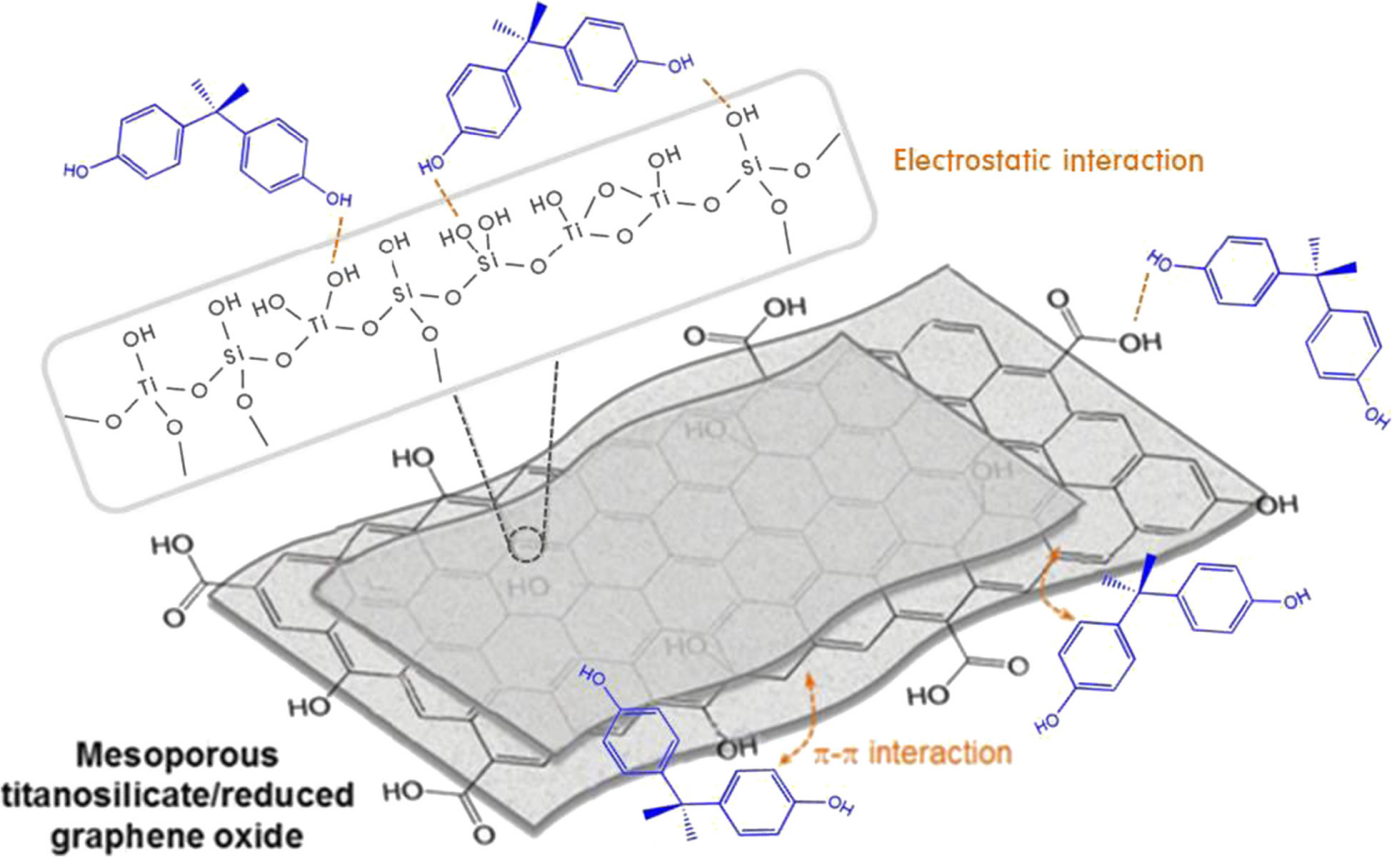
An illustration for the adsorption interaction of BPA with mesoporous titanosilicate/reduced graphene oxide composite materials.

Figure 7 illustrates the adsorptive interaction of BPA with adsorption sites of mesoporous titanosilicate/reduced graphene oxide composite materials. BPA adsorbs onto mesoporous titanosilicate/reduced graphene oxide composite materials by either electrostatic interaction between the hydroxyl groups contained in BPA and the hydroxyl groups on the adsorbents or the π-π interaction between the benzene rings of BPA and the sp^2^ domains of the reduced graphene oxide. In the case of the electrostatic interaction, the hydroxyl groups on the adsorbents can be divided into three different types of adsorbent sites, namely, OH on the reduced graphene oxide, OH bonded to Si atoms, and OH bonded to Ti atoms. However, it was observed that the adsorption amount was essentially proportional to the surface area of the composite materials.

## Conclusions

We prepared mesoporous titanosilicate/reduced graphene oxide composite materials with different Ti contents and used them as adsorbents for removing BPA from a water system. Adsorption amounts acquired from adsorption kinetics are inversely proportional to the Ti content since the surface areas of the composite materials decreased with increasing Ti content. In this study, the adsorptive interaction of BPA with the adsorbents was observed by FT-IR and Raman analyses. From the observation, we reached the conclusion that BPA is adsorbed onto the composite materials through two different interactions: (i) electrostatic interactions between the hydroxyl groups of BPA and mesoporous titanosilicate or reduced graphene oxide and (ii) π-π interactions between the benzene rings of BPA and the sp^2^ domains of reduced graphene oxide.

## Competing interests

The authors declare that they have no competing interests.

## Authors’ contributions

NK performed the adsorption tests and drew the figures. CNH characterized the samples with Raman and FT-IR. TDNP designed the experiments and synthesized the samples. IKY was involved in the writing process. EWS designed the experiments and wrote the manuscript. All authors have read and approved the final manuscript.
